# The Factors Influencing Feeding Practices of Primary Caregivers of Preschoolers: A Theory-Based Cross-Sectional Study

**DOI:** 10.3390/children12020226

**Published:** 2025-02-13

**Authors:** Qutaibah Oudat, Rebecca C. Lee, Elaine L. Miller, Sarah Collins Couch, Tamilyn Bakas

**Affiliations:** 1 Department of Population Health, College of Nursing, University of Cincinnati, Cincinnati, OH 45221, USA; lee2rc@ucmail.uc.edu (R.C.L.); millerel@ucmail.uc.edu (E.L.M.); bakastn@ucmail.uc.edu (T.B.); 2 Department of Rehabilitation, College of Allied Health Sciences, University of Cincinnati, Cincinnati, OH 45221, USA; couchsc@ucmail.uc.edu

**Keywords:** comprehensive feeding practices, theory of planned behavior, preschoolers, caregivers, parents

## Abstract

Background/Objectives: Primary caregivers play a pivotal role in shaping children’s dietary behaviors, which are critical in preventing childhood obesity. This study aimed to examine the extent to which demographic characteristics and caregiver factors, including dietary beliefs and intentions to provide a healthy diet, influence feeding practices among primary caregivers of preschool-aged children. Methods: This study included a cross-sectional dataset comprising data collected from 146 primary caregivers of preschool-aged children (3–5 years). Participants were recruited using snowball and convenience sampling from Facebook and community settings in Cincinnati, Ohio. Feeding practices were assessed using the Comprehensive Feeding Practices Questionnaire (CFPQ), while caregiver demographic characteristics, dietary beliefs, and intentions were measured through self-administered surveys. Hierarchical multiple linear regression was employed to identify predictors of feeding practices. Results: The findings reveal that primary caregivers’ feeding practices were shaped by multiple factors, including dietary beliefs (behavioral, normative, and control), intentions, and demographic characteristics. Each subscale of feeding practices was predicted by different factors, highlighting the unique influence of these factors on feeding behaviors. Conclusions: This study underscores the complex interplay between caregiver cognitive (dietary beliefs and intentions) and demographic factors in shaping feeding practices. The findings highlight the importance of targeting caregiver beliefs and intentions in interventions aimed at promoting healthier feeding practices, contributing to the prevention of childhood obesity. Importantly, future research is needed to explore these factors in more diverse populations and validate cognitive measures for broader application.

## 1. Introduction

The prevalence of childhood obesity has risen dramatically over the past few decades, with significant adverse health and economic consequences [1,2,3]. In the United States, one in three adults and one in six children are affected by obesity, a condition linked to chronic illnesses and premature mortality [4]. According to the 2023 report of the World Obesity Atlas, the expected annual increase in obesity between 2020 and 2030, if untreated, is 2.1% and 2.4% for adults and children, respectively [3].

While the etiology of obesity is complex and multifactorial [5], evidence highlights the critical role of parental feeding practices in influencing children’s weight outcomes and eating behaviors, particularly during the early years of life [6,7]. For instance, a systematic review by Shloim et al. (2015) including caregivers with their young children found that feeding practices, such as modeling healthy behaviors and encouraging balanced eating, were consistently associated with a healthy BMI in children. On the other hand, a lack of parenting involvement in monitoring diet and minimal interaction during meals was linked to a higher BMI and unhealthy dietary habits in children [8]. Similarly, other feeding practices, such as restriction and pressure practices, were also found to be associated with adverse weight outcomes in young children [8,9]. These practices can disrupt the self-regulation of energy intake and increase the risk of overeating when such foods become available.

A growing body of evidence has shown that the feeding practices of caregivers can vary depending on various factors, such as personal characteristics, cultural norms, socioeconomic conditions, psychological stressors, and the broader food environment [10,11,12]. Studies, for example, showed that the caregivers who perceived their child to be obese were more likely to use monitors and restrictions for weight control practices, whereas those who perceived their child to be underweight, adopted pressure feeding practices to encourage their child to eat more food [13,14]. Other studies have also indicated that high and uncontrolled parental stress was significantly associated with less desirable feeding practices, such as food as a reward, pressure to eat, and emotional regulation [15,16]. However, current research provides limited insight into the influence of cognitive factors, such as beliefs and intentions, on the feeding practices caregivers adopt with their children. This might limit our understanding of the underlying mechanisms that significantly shape specific feeding practices, particularly in the context of beliefs, attitudes, and intentions. The purpose of this study was to utilize the constructs of the Theory of Planned Behaviors (TPB) [17] to determine how demographic characteristics and caregiver cognitive factors, such as dietary beliefs and intentions, contribute to variations in feeding practices among primary caregivers of preschool-aged children. The TPB is a cognitive theory that focuses on cognitive processes that influence an individual’s intentions and subsequent behaviors [17]. Our study is among the first studies that apply a structured theoretical framework to better understand the determinants of caregiver feeding practices. The findings from this study can inform the development of targeted interventions and educational programs designed to promote healthier feeding practices, ultimately contributing to the prevention of childhood obesity and the establishment of lifelong healthy eating habits in preschool-aged children.

## 2. Materials and Methods

### 2.1. Study Design

**Figure 1:**
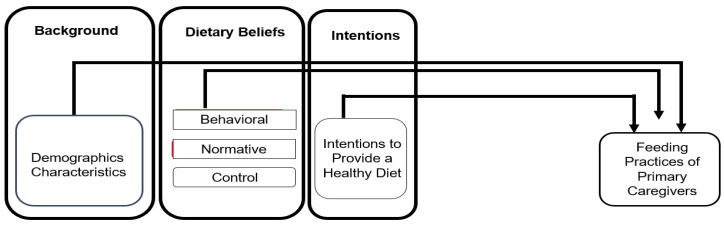
Conceptual model.

Guided by a conceptual model derived from the TPB (Figure 1), this study is a cross-sectional study aimed at exploring how demographic characteristics and primary caregiver dietary beliefs and intentions contribute to variations in feeding practices in primary caregivers of preschool-aged children. In this study, a primary caregiver is defined as an unpaid individual (e.g., a father, mother, or grandparent) who is responsible for managing and arranging meals for a child aged 3 to 5 years old. The reporting of this study follows the Strengthening Reporting of Observational Studies in Epidemiology (STROBE) guidelines to ensure clarity and transparency [18].

### 2.2. Sample Size, Study Population, and Eligibility

The data used in this study were collected as part of our previous study [19] to determine the extent to which the demographic characteristics and the factors of primary caregivers (dietary beliefs, intention to provide a healthy diet, feeding practices) can explain the variance in the diet quality of preschoolers.

The sample size (n = 146) was calculated based on Green’s regression formula [20], ensuring sufficient power to detect significant associations in the regression analysis. The study population was primary caregivers of children aged 3 to 5 years residing in the United States. Primary caregivers were individuals responsible for the child’s daily care, including the preparation and management of the child’s meals. Inclusion criteria required caregivers to (1) be at least 18 years old, (2) be able to read and talk in English, (3) be unpaid primary caregivers of a child aged 3–5, (4) be responsible for managing and arranging meals, (5) have free access to a telephone, (6) have no hearing or speaking difficulties, (7) be caring for a preschool-aged child without serious psychiatric or mental impairments or conditions requiring a special diet, and (8) have sufficient time to voluntarily participate in the study. Caregivers who were pregnant were excluded from participation due to the potential impact of pregnancy on maternal feeding practices.

### 2.3. Sampling Techniques and Study Settings

Data in this study were collected using multiple sampling techniques (i.e., snowball and convenience) to facilitate quick and accessible participant recruitment. Participants were recruited from Facebook (www.Facebook.com) [21] and various settings and facilities in Midwest (1) daycare and early childhood care and education centers and (2) community religious institutions (i.e., Mosques, Churches, and Synagogues). Data were collected between April 2022 and March 2023, through caregiver-completed surveys and dietary assessments of preschool-aged children.

### 2.4. Study Procedure and Protocol

The study flyer was disseminated through a nine-month Facebook advertising campaign and posted in newsletters and bulletins across study facilities in the Midwest. The flyer invited interested primary caregivers to participate by (1) clicking a Facebook link, (2) scanning a barcode, or (3) contacting the principal investigator (PI) directly. Participants who used the Facebook link or barcode completed an online study postcard and eligibility checklist, while those who contacted the PI were guided through these steps over the phone. Eligible participants were contacted to confirm their eligibility, address questions, and obtain consent. Study materials were then provided via email (using a secure Redcap link) or mailed to participants. Participants who received the study package by mail were provided additional instructions to mail the completed package to the PI.

The study package included an information sheet and three self-administered questionnaires (SAQ). The information sheet detailed the study’s objectives, procedures, and details about incentives. No financial compensation was provided to participants. The SAQs assessed demographic data, dietary beliefs, intentions to provide a healthy diet, and feeding practices. The SAQ took 20–30 min to complete.

### 2.5. Measurements and Variables

Primary Outcome. The primary outcome of this study was the feeding practices of caregivers, measured using the Comprehensive Feeding Practices Questionnaire (CFPQ). The CFPQ involved 49 items measuring 12 practices subscales, such as (1) child control, (2) modeling, and (3) monitoring. The items of each subscale were rated using a 5-point Likert scale. The total score of each subscale was calculated, with higher scores indicating a greater frequency of feeding practices [22].

Predictor Variables. The predictor variables included demographic characteristics and primary caregiver variables, including dietary beliefs (i.e., behavioral, normative, and control beliefs) and intentions to provide a healthy diet. Participants reported their age, sex, gender, education level, smoking status, marital status, and working status. Data on their child’s age, sex, and gender were also collected. The heights and weights of both participants and their children were estimated to calculate BMI using the imperial formula (weight in pounds (lb.)/[height in inches (in)]^2^ × 703) [23].

The other variables including dietary beliefs and intentions to provide a healthy diet were also collected through a self-reported questionnaire. These variables were measured using a questionnaire developed based on TPB, and items of each construct were rated based on a Likert scale. Behavioral beliefs were assessed with 11 items on a 7-point Likert scale (“extremely unlikely” to “extremely likely”), evaluating perceived outcomes of providing a healthy diet to preschool-aged children. Normative beliefs were measured with 4 items on the same scale, assessing perceived social support. Control beliefs were evaluated with 12 items on a 7-point scale (“very rarely” to “very frequently”), examining factors influencing the provision of a healthy diet. Intentions to provide a healthy diet were assessed with a single item (“unlikely” to “likely”), measuring participants’ readiness.

### 2.6. Statistical Methods

Data were analyzed in IBM SPSS Statistics (Version 28) [24]. Missing values were assessed, and data with ≥25% missing observations were excluded from analyses [25]. Descriptive statistics were used to summarize the variables, and regression assumptions (i.e., normality of residuals, linearity, homoscedasticity, and multicollinearity) were assessed for each regression model. Cronbach’s (internal consistency) was measured for all variables, including outcome and predictors.

Guided by the study’s conceptual framework, hierarchical multiple linear regression (enter model) was chosen for its ability to assess the contribution of predictor variables at each step of the analysis [26]. Pearson correlation, independent samples t-tests, and ANOVA were initially used to identify variables significantly associated with feeding practices (*p* < 0.05). Only variables showing a significant association with the feeding practices score (*p* < 0.05) were included in the regression model. Significant nominal variables were transformed into dummy variables (0/1) for inclusion in the regression models.

## 3. Results

Table 1 summarizes the demographic characteristics of the primary caregivers included in this study. This study included 146 primary caregivers (biological mothers 88.4%, adoptive mothers 4.1%, biological fathers 4.1%, stepmothers 1.4, and grandmothers 2.1) who met the eligibility criteria and provided completed data. The average age of the caregivers is 35.4 years, with a mean BMI of 32.2. Most caregivers are female (95.9%) and predominantly identify as White/Caucasian (77.4%). Educational attainment is high, averaging 16.4 years. In terms of employment, 34.2% work full-time, while others are part-time (19.9%) or self-employed (19.2%). Income levels vary, with 24.7% reporting household earnings above USD 100,000. A significant proportion (56.2%) expressed concern about their weight, yet only 19.9% follow a diet. Additionally, 45.2% perceive their household income as comfortable, while 47.3% indicate they are just making ends meet.

The preschool-aged children of primary caregivers in this study had a mean age of 3.9 years (SD = 0.8) and a BMI of 17.5 kg/m^2^ (SD = 6.6). Also, nearly half of the children were identified as female (49.3%), 50.0% as male, and 0.7% as “other.” Similarly, 50.0% of the children were identified as biologically male and 50.0% as female.

As reported in our previous study [19], Cronbach’s alpha (*α*) was calculated for all multi-item subscales to assess their internal consistency. The internal consistency of the Dietary Beliefs subscales was good, with Cronbach’s alpha values of 0.83 for Behavioral and 0.89 for Normative beliefs, indicating reliable measurement of caregivers’ dietary perceptions. The Control subscale also showed acceptable consistency, with alpha values of 0.71 for “Facilitators” and 0.78 for “Hindrances.” For the feeding practices of caregivers (12 subscales), most subscales demonstrated acceptable reliability (α ≥ 0.70), but a few subscales, such as “Encourage Balance and Variety” (α = 0.56), “Environment” (α = 0.34), and “Teaching About Nutrition” (α = 0.31), exhibited lower internal consistency. However, all subscales were retained to provide a comprehensive assessment of feeding practices, but findings related to them should be interpreted cautiously. Supplementary Table S1 summarizes the mean ± SD, range, and Cronbach’s alpha (*α*) of the predictors and primary outcomes.

### 3.1. Correlation of Demographic Characteristics with the Feeding Practice Subscales of Primary Caregivers

The Pearson correlation coefficient (r) revealed significant relationships between caregiver and child demographic characteristics, including age, BMI, and years of education with various feeding practices. Caregiver age showed a weak negative correlation with teaching about nutrition (r= −0.2), while child age had a positive correlation with restriction for health (r= 0.2). Caregiver BMI was negatively correlated with emotion regulation (r = −0.2), and child BMI was positively correlated with restriction for weight control (r = 0.2). Caregiver years of education were positively correlated with emotion regulation practices (r = 0.2) and negatively correlated with encouraging balance variety and restriction for health (r = −0.2).

Additionally, the results of the t-test showed significant Mean (M) differences in feeding practices based on caregiver gender, race, and following a specific diet. Male caregivers had a significantly lower degree of restriction for weight control practice compared to female caregivers, with a mean difference of 3.9. Non-White racial groups had significantly lower scores in monitoring, restriction for health, and restriction for weight control feeding practices, with a mean difference of approximately 2.2 compared to White caregivers. Caregivers who followed a specific diet scored significantly higher on environment feeding practices, with a mean difference of 2.8 compared to those who did not follow any diet.

Lastly, the ANOVA further revealed significant mean differences in feeding practices across various demographic and socioeconomic factors. Marital status emerged as an important factor, with differences observed in environment feeding practices (F = 3.3, *p* < 0.05) and restrictions for health (F = 3.9), suggesting that marital dynamics may influence caregiving behaviors related to the feeding environment and health-related restrictions. Smoking status was also associated with feeding behaviors, as significant differences were identified in involvement feeding practices (F = 2.9), indicating that smoking habits may impact the level of caregiver engagement in feeding. Working status played a notable role, with significant differences found in involvement (F = 3.5) and restrictions for health (F = 3.5) feeding practices, reflecting the potential influence of occupational responsibilities on caregiving strategies. Similarly, household income was associated with modeling feeding practices, with significant variations observed across different income levels (F = 3.8), highlighting the impact of socioeconomic conditions on feeding behaviors. The caregiver’s relationship to the preschool-aged child, such as whether they were a parent or grandparent, significantly influenced the use of food as a reward (F = 2.5). Further, caregivers who were more concerned about their child’s weight demonstrated differences in environment feeding practices (F = 5.6), suggesting that weight-related concerns may shape the feeding environment. Perceptions of the child’s weight further influenced feeding behaviors, with significant differences in environment feeding practices (F = 4.03) and restrictions for weight control (F = 3.7). Finally, caregivers’ perceptions of their own weight were significantly associated with control feeding practices (F = 4.2) and emotion regulation feeding practices (F = 2.5), indicating that self-perceptions shape how caregivers manage dietary behaviors.

Overall, demographic factors that had a significant correlation were added to the final analysis model. Table 2 summarizes the demographic characteristics that were significantly associated with the feeding practices of caregivers. The findings highlight the complex interplay between caregiver demographics, socioeconomic conditions, and perceptions in shaping feeding practices. The results emphasize the influence of relational dynamics, financial stability, and weight-related concerns on feeding behaviors, particularly in areas related to environment, restrictions, and control.

### 3.2. Correlation Between Dietary Beliefs and Intentions with the Feeding Practice Subscales of Primary Caregivers

Table S1 (Supplementary Materials) highlights the association between dietary beliefs, intentions, and feeding practice subscales of primary caregivers. Only factors that revealed a significant association were included in the final analysis model. Using Pearson correlation coefficients, the findings revealed several significant correlations with different feeding subscales.

Behavioral beliefs, which reflect caregivers’ attitudes and perceptions a person holds about the likely outcomes or consequences of a particular behavior, were significantly associated with several feeding practices. Stronger behavioral beliefs were linked to less controlling feeding practices, as indicated by a negative correlation (r = −0.24). These beliefs also showed positive associations with practices such as encouraging balance and variety (r = 0.23), fostering a supportive feeding environment (r = 0.17), and using food as a reward (r = 0.21). Furthermore, behavioral beliefs were moderately associated with modeling healthy eating behaviors (r = 0.26) and teaching children about nutrition (r = 0.33), underscoring the role of these beliefs in promoting educational and proactive feeding strategies. Positive correlations with monitoring (r = 0.23), restriction for health and pressure (r = 0.21), and restriction for weight control (r = 0.17) suggest that caregivers with stronger behavioral beliefs also adopt practices aimed at oversight and health management. Collectively, these findings illustrate that behavioral beliefs significantly shape a range of feeding practices, from educational approaches to strategies that manage dietary behaviors.

Normative beliefs, reflecting caregivers’ perceptions of socially expected feeding behaviors, also influenced feeding practices, although to a lesser extent. Significant correlations were observed with encouraging balance and variety (r = 0.20), modeling (r = 0.24), and teaching about nutrition (r = 0.19). These associations suggest that caregivers who internalize normative dietary expectations are more likely to engage in practices that promote variety, healthy modeling, and nutritional education. Although weaker than behavioral beliefs, normative beliefs still play an important role in shaping positive feeding behaviors.

Control facilitators, which measure caregivers’ perceived ability to manage feeding, demonstrated strong associations with several feeding practices. Caregivers with greater perceived control were more likely to encourage balance and variety in diets (r = 0.217), involvement (r = 0.32), and model healthy eating behaviors (r = 0.27). Significant correlations were also observed with monitoring (r = 0.32) and restriction for weight control (r = 0.22), highlighting the proactive and health-focused strategies employed by caregivers who feel confident in their ability to manage feeding. Conversely, caregivers with greater perceived control were less likely to use control practices (r= −0.174). These findings underscore the importance of caregiver self-efficacy in promoting positive and structured feeding practices.

On the other hand, hindrances to control, which capture barriers to effective feeding, revealed a more complex pattern of associations. Caregivers who perceived more barriers were less likely to engage in practices such as encouraging balance and variety (r = −0.17) and modeling (r = −0.21). However, these hindrances were positively correlated with practices like emotion regulation (r = 0.226), food as a reward (r = 0.24), and restriction for health (r = 0.217), suggesting that caregivers facing challenges may turn to strategies aimed at managing emotional or health-related aspects of feeding. This duality highlights the nuanced role of perceived barriers in influencing caregiving behaviors.

Intentions to provide a healthy diet emerged as the strongest driver of positive feeding practices. Caregivers with stronger intentions demonstrated less controlling feeding practices (r = −0.20) and were significantly more likely to encourage balance and variety (r = 0.44), model healthy eating behaviors (r = 0.33), monitor dietary habits (r = 0.24), and teach about nutrition (r = 0.22). These findings underscore the pivotal role of caregivers’ intentions in fostering feeding behaviors that align with healthy dietary outcomes. Intentions appear to serve as a motivational foundation for engaging in proactive, supportive, and educational feeding practices.

### 3.3. Key Predictors of Caregivers’ Feeding Practices

All regression models for the 12 CFPQ subscales met the required assumptions for valid analysis. Residuals for each model were approximately normally distributed, as confirmed by Q-Q plots and the Shapiro–Wilk test (*p* > 0.05). Linearity and homoscedasticity were demonstrated by scatterplots of residuals versus predicted values, which showed no significant deviations. Multicollinearity was not detected, with all Variance Inflation Factors (VIF) below 10. Additionally, the Durbin–Watson test values ranged between 1.6 and 2.4 for all models, indicating no evidence of autocorrelation in residuals. These findings support the validity of the regression analyses in explaining variations in feeding practices as measured by the CFPQ subscales.

Table 3 presents the final models from the hierarchical multiple linear regression analyses (enter method), which highlighted the key predictors of the 12 feeding practice subscales and how much these predictors together explain the variance in each subscale.

- Control Feeding Practice. Among the predictors included in the regression model, caregivers who perceived their weight as normal (β = 0.202, *p* = 0.012) demonstrated a positive association with control feeding practice scores. This suggests that caregivers with a normal weight perception may feel more confident in their feeding practices, possibly reflecting in allowing their children to control or make decisions regarding their food choices. In contrast, caregivers unable to perceive their weight (categorized as “don’t know”) were significantly associated with lower control feeding practice scores (β = −0.198, *p* = 0.011), indicating higher control over the food selection of their preschooler child. Additionally, behavioral beliefs (β = −0.196, *p* = 0.017) and intentions to provide a healthy diet (β = −0.167, *p* = 0.036) were significant predictors. The negative associations suggest that caregivers with strong beliefs and intentions about specific feeding behaviors may adopt less controlling approaches. Overall, these predictors explained 20.2% of the variance in control feeding practices (R^2^ = 0.202, Adjusted R^2^ = 0.162).
- Emotion Regulation Feeding Practice. Of the predictors included in this model, only hindrances control belief emerged as a significant predictor of the emotion regulation feeding practice subscale (β = 0.294, *p* = 0.004). The regression model explained 16.1% of the variance in emotion regulation feeding practices (R^2^ = 0.161, Adjusted R² = 0.118). This suggests that caregivers who perceive greater barriers or challenges in their control beliefs were more likely to engage in these practices.
- Encourage Balance and Variety. Of the predictors included in this model, years of education and intention to provide a healthy diet were significant predictors of the encourage balance and variety feeding practice subscale. Years of education was negatively associated with this subscale (β = −0.172, *p* = 0.020), while intention to provide a healthy diet showed a strong positive association (β = 0.374, *p* < 0.001). Together, these predictors explained 28.4% of the variance in encourage balance and variety feeding practices (R^2^ = 0.284, Adjusted R^2^ = 0.253). This suggests that caregivers with higher intentions to provide a healthy diet were more likely to encourage balance and variety, whereas higher levels of education may be associated with less emphasis on this feeding practice.
- Environment Feeding Practices. Of the predictors included in this model, following a diet, divorced marital status, and behavioral beliefs were significant predictors of the environment feeding practice subscale. Following a diet (β = −0.164, *p* = 0.047) and divorced marital status (β = −0.186, *p* = 0.049) were negatively associated with this subscale, while behavioral beliefs showed a positive association (β = 0.159, *p* = 0.020). Together, these predictors explained 20.4% of the variance in environment feeding practices (R^2^ = 0.204, Adjusted R^2^ = 0.145). This indicates that caregivers who followed a specific diet or were divorced may place less emphasis on environmental feeding practices, whereas stronger behavioral beliefs were linked to a greater focus on shaping the feeding environment.
- Food as a Reward Feeding Practices. Of the predictors included in this model, being a grandmother and behavioral beliefs were significant predictors of the food as a reward feeding practice subscale. Being a grandmother was negatively associated with this subscale (β = −0.212, *p* = 0.010), while behavioral beliefs showed a positive association (β = 0.208, *p* = 0.009). Together, these predictors explained 10.9% of the variance in food as a reward feeding practices (R^2^ = 0.109, Adjusted R^2^ = 0.077). This suggests that grandmothers were less likely to use food as a reward, whereas stronger behavioral beliefs were associated with a more likelihood of employing this practice.
- Involvement Feeding Practices. Of the predictors included in this model, smoking status and behavioral beliefs were significant predictors of the involvement feeding practice subscale. Smoking status was negatively associated with this subscale (β = −0.157, *p* = 0.046), while behavioral beliefs showed a strong positive association (β = 0.301, *p* < 0.001). Together, these predictors explained 24.0% of the variance in involvement feeding practices (R^2^ = 0.240, Adjusted R^2^ = 0.190). This indicated that caregivers who smoke were less likely to be involved in feeding practices, whereas stronger behavioral beliefs were linked to greater involvement.
- Modeling Feeding Practice. Of the predictors included in this model, comfortable income, behavioral beliefs, hindrance control beliefs, and intentions to provide a healthy diet were significant predictors of the modeling feeding practice subscale. Comfortable income (β = 0.168, *p* = 0.006) and behavioral beliefs (β = 0.230, *p* = 0.030) were positively associated with this subscale, while hindrance control beliefs showed a negative association (β = −0.218, *p* = 0.008). Intentions to provide a healthy diet also demonstrated a positive association (β = 0.216, *p* = 0.005). Together, these predictors explained 28.0% of the variance in modeling feeding practices (R^2^ = 0.280, Adjusted R^2^ = 0.244). These findings suggest that caregivers with a comfortable income, strong behavioral beliefs, and intentions to provide a healthy diet were more likely to model feeding practices, whereas perceiving more hindrances in control beliefs was associated with a lower likelihood of modeling.
- Monitoring Feeding Practice. Of the predictors included in this model, facilitators’ control beliefs and intentions to provide a healthy diet were significant predictors of the monitoring feeding practice subscale. Facilitators’ control beliefs showed a positive association with this subscale (β = 0.222, *p* = 0.025), as did intentions to provide a healthy diet (β = 0.178, *p* = 0.009). Together, these predictors explained 16.4% of the variance in monitoring feeding practices (R^2^ = 0.164, Adjusted R^2^ = 0.140). These results suggest that caregivers with stronger facilitators’ control beliefs and greater intentions to provide a healthy diet were more likely to engage in monitoring feeding practices.
- Pressure Feeding Practice. Of the predictors included in this model, behavioral beliefs were the only significant predictor of the pressure feeding practice subscale. Behavioral beliefs showed a positive association with this subscale (β = 0.209, *p* = 0.012). This model explained 4.3% of the variance in pressure feeding practices (R^2^ = 0.043, Adjusted R^2^ = 0.037). These findings suggest that caregivers with stronger behavioral beliefs were more likely to apply pressure in their feeding practices.
- Restriction for Health Feeding Practice. Of the predictors included in this model, child age, employment status (not working), and hindrance control beliefs were significant predictors of the restriction for health feeding practice subscale. Child age (β = 0.202, *p* = 0.001), employment status (not working; β = 0.248, *p* = 0.008), and hindrance control beliefs (β = 0.252, *p* = 0.003) all showed positive associations with this subscale. Together, these predictors explained 30.9% of the variance in restriction for health feeding practices (R² = 0.309, Adjusted R^2^ = 0.241). These results suggest that older children, caregivers who were not working and perceived greater hindrances in control beliefs were more likely to restrict feeding for health reasons.
- Restriction for Weight Control Feeding Practice. Of the predictors included in this model, being a stepmother was a significant predictor of the restriction for weight control feeding practice subscale. Being a stepmother showed a positive association with this subscale (β = 0.210, *p* = 0.014). This model explained 29.4% of the variance in restriction for weight control feeding practices (R^2^ = 0.294, Adjusted R^2^ = 0.236). These findings suggest that stepmothers were more likely to engage in feeding practices that involve restriction food for weight control.
- Teaching about Nutrition Feeding Practice. Of the predictors included in this model, the age of the primary caregivers, behavioral beliefs, and intentions to provide a healthy diet were significant predictors of the teaching about nutrition feeding practice subscale. The age of the primary caregivers was negatively associated with this subscale (β = −0.169, *p* = 0.023), while behavioral beliefs (β = 0.283, *p* = 0.001) and intentions to provide a healthy diet (β = 0.182, *p* = 0.032) showed positive associations. Together, these predictors explained 16.9% of the variance in teaching about nutrition feeding practices (R^2^ = 0.169, Adjusted R^2^ = 0.145). These results suggest that younger caregivers, stronger behavioral beliefs, and greater intentions to provide a healthy diet were associated with greater engagement in teaching about nutrition.

## 4. Discussion

Guided by a framework derived from the TPB, this study was among the first studies to investigate how demographic characteristics and caregiver factors (dietary beliefs and intentions) contribute to variations in feeding practices among primary caregivers of preschool-aged children. Feeding practices were assessed using the CFPQ which is a validated tool designed to measure the twelve dimensions of caregiver feeding practices. To our knowledge, existing studies have mainly focused on the impact of demographic characteristics without considering the cognitive decision-making processes of caregivers that can significantly impact their feeding practices. The hierarchical multiple linear regression analyses revealed that the variance in each feeding practice subscale was explained by different predictors (i.e., demographic characteristics, dietary beliefs, and intentions to provide a healthy diet). Overall, the findings highlight the complex interplay between caregivers’ beliefs, intentions, and sociodemographic factors in shaping feeding practices of primary caregivers of preschoolers.

The findings of our study align with the existing literature [27,28,29,30] on the influence of demographic characteristics on caregiver feeding behaviors and beliefs. Key demographic factors, including weight perception, education, income, and marital status, significantly shape feeding practices, highlighting the complex interplay of individual, social, and environmental determinants in shaping the feeding practices of primary caregivers. For instance, our study indicated that caregivers who perceived their weight as “normal” were more likely to allow their preschool-aged children to control or make decisions regarding their own food choices. This finding has also suggested that uncertainty about their weight status was linked to a more controlling or restrictive approach to feeding, where caregivers feel less confident in granting their child independence in food choices. A lack of awareness about one’s own weight could translate into lower trust in a child’s ability to self-regulate their eating behaviors, potentially leading to more directive feeding practices. Existing studies have limitedly studied the impact of the primary caregivers’ weight on their feeding strategy. Previous studies [31,32,33] have consistently indicated that parents’ perception of their child’s weight status affects their feeding practices, with restriction being a common response to perceived overweight.

Importantly, our study demonstrated a distinct pattern of influence, highlighting differences compared to previous findings. For instance, while our study revealed that the year of education was inversely associated with encouraging balance and variety of food, other studies [29,34] found that caregivers with higher education and income were more likely to adopt health-promoting feeding strategies, such as encouraging variety and balance in meals. Similarly, a study by Trevino et al. (2021) [35] underscored the gender differences in how feeding practices mediate emotional eating, with mothers and fathers showing distinct patterns. The study found that mothers (with a child aged between 5 and 13) primarily relied on restrictive feeding practices to mediate the relationship between their emotional eating and their child’s emotional eating [35]. Interestingly, our study found that being a mother was not significantly associated with any feeding practices. These discrepancies in results might be attributed to several factors, including differences in study populations, instruments, methodologies, and the age groups targeted.

Along with the findings of our study, previous studies [36,37,38] have highlighted the significant influence of caregivers’ intentions, needs, and socioeconomic factors on feeding practices. These studies suggest that caregivers’ strong intentions to provide a healthy diet are directly associated with adopting responsive feeding practices (e.g., emotion regulation, encouraging balance and variety, and involvement), which can help to promote autonomy and healthy eating behaviors by responding to a child’s hunger and satiety cues in a supportive manner. Additionally, results from previous studies have consistently demonstrated the importance of dietary beliefs (e.g., parental knowledge) to promote healthier feeding practices [39,40,41]; our study revealed that the behavioral beliefs (which reflect a caregiver’s perceived knowledge about the consequences of following dietary behavior) can impact various feeding practices subscales. For instance, when caregivers hold positive attitudes and expectations regarding the benefits of adhering to a healthy diet, they are more likely to engage in responsive feeding practices. Conversely, negative or uncertain perceptions about dietary behaviors may contribute to less favorable feeding approaches. However, since our study did not assess whether caregivers’ beliefs about following a healthy diet were positive or negative, future research is needed to explore how these varying perceptions may differentially influence feeding practices and dietary outcomes. Additionally, future studies are needed to evaluate the primary caregivers diet literacy prior to any future interventions. Furthermore, while normative beliefs were not associated with any feeding subscales in this study, existing studies [39,40] have emphasized the significant impact of “normative culture” on feeding practices. For example, a study by Lindsay et al. (2020) [42] on paternal feeding practices has shown that while fathers recognized the value of nutritious diets, they sometimes allowed their children to eat less healthy foods as a way to bond with their children, reward them, or avoid conflict during mealtimes. However, the link between social norms and feeding practices continues to be understudied.

Consistent with other studies [42,43], our study has also revealed a significant link between control beliefs (hindrance and facilitator) and different feeding subscales. For example, hindrance control beliefs (including limited financial resources, conflicting work schedules, and safety concerns about neighborhood) can lead to adopting pressurized feeding (e.g., restriction for health, emotion regulation). Conversely, facilitator control beliefs, such as having reliable access to nutritious food and a supportive caregiving environment, may encourage responsive feeding practices (e.g., monitoring and modeling) that can promote healthier eating behaviors in children. Overall, these findings underscore the critical role of parental control beliefs in shaping feeding strategies and highlight the importance of addressing external barriers to foster positive dietary habits.

### 4.1. Strengths and Limitations

This study is among the first studies to apply the TPB construct to uncover how caregiver intentions and behavioral beliefs shape the feeding practices of caregivers with preschool-aged children (3–5 years). The application of the TPB could advance the literature by integrating demographic and psychological predictors to explain variations in feeding behaviors. This study has also utilized the CFPQ, a validated and widely used instrument that captures a broad spectrum of feeding practices. This comprehensive assessment enables a nuanced understanding of caregiver feeding practices, contributing to the reliability and validity of the findings. Another strength of this study is the use of diverse sampling techniques, which enhance the representativeness of the findings.

However, our study also holds limitations that we will elaborate on further. First, the cross-sectional design precludes causal inferences, limiting the ability to establish temporal relationships between predictors and feeding practices. Additionally, the analysis included 12 dependent variables of the CFPQ, increasing the risk of Type I errors (rejects a null hypothesis that is actually true). Second, while the TPB is a well-established theoretical framework, the instrument used to measure its constructs (behavioral beliefs, normative beliefs, control beliefs, and intentions) in this study has not been previously validated. The lack of established psychometric properties can raise concerns about the accuracy and robustness of the findings. Additionally, the majority of participants in this study identified as White/Caucasian, resulting in a relatively homogeneous sample. This lack of ethnic and cultural diversity may limit the external validity and generalizability of the findings to more diverse populations, particularly those with varying sociocultural norms and practices regarding feeding behaviors. Third, the use of self-administered surveys for data collection introduces potential biases that could impact the accuracy of the results. Recall bias may have occurred, as participants might have struggled to accurately report past behaviors or attitudes. Moreover, social desirability bias and the Hawthorne effect may have influenced participants to provide responses that align with perceived societal norms or expectations rather than their actual practices. These biases could result in an overestimation of positive behaviors or the underreporting of less desirable ones. Finally, variations in participants’ sociodemographic characteristics, such as educational attainment or literacy levels, may have contributed to misunderstandings of some of the survey questions or instructions. This could lead to inaccurate responses and reduce the reliability of the data.

### 4.2. Future Directions

This study outlines a framework derived from the TPB to investigate factors affecting the feeding practices of primary caregivers of preschoolers in the US. The results of this study provide preliminary data, analysis, and the template for future studies that are aimed at understanding the complex interplay between caregivers’ beliefs, intentions, and demographic characteristics in shaping children’s dietary habits. While this study relied on an instrument that was adaptive from the TPB to measure dietary beliefs and intentions, future studies are needed to develop and validate an instrument to measure these constructs, enhancing the accuracy and generalizability of findings in future investigations.

Further investigations are also essential to expand upon these findings by exploring additional psychosocial and environmental factors that may influence caregivers’ feeding practices. Future studies should expand the framework of this study by integrating additional theoretical models and constructs that account for broader influences on caregivers’ feeding practices. While this study emphasized caregiver-specific factors (beliefs and intentions), broader environmental influences, such as food accessibility, advertising, and policy, were not addressed. Longitudinal studies might also be essential to explore the temporal and causal relationships between caregiver characteristics, beliefs, intentions, with the feeding practice subscales. Such designs will provide critical insights into how feeding practices evolve over time, as children grow, and caregivers adapt to their developmental needs. Importantly, recruiting ethnically and culturally diverse participants in future studies is vital for understanding how cultural norms, values, and socioeconomic factors influence feeding behaviors. These efforts together will support the development of culturally sensitive interventions tailored to diverse populations. By addressing these directions, future studies can deepen our understanding of caregiver feeding practices and contribute to the design of effective, evidence-based interventions for promoting healthy feeding practices and improving the diet quality of young children.

### 4.3. Implications for Practice

This study highlights caregiver feeding practices as a critical component of improving the diet quality of young children and preventing obesity in early childhood. These findings emphasize the importance of targeting caregivers’ cognitive factors, such as beliefs and intentions, in designing interventions to promote healthier feeding practices. Tailored programs integrated into parenting education or early childhood care can enhance caregivers’ self-efficacy and address socioeconomic and cultural barriers. Clinically, the CFPQ, utilized in this study, could be integrated into routine pediatric assessments. This tool would allow healthcare providers to systematically identify areas where caregivers may need additional support, such as improving their involvement in feeding or reducing reliance on restrictive practices. Importantly, educational programs for caregivers should emphasize the importance of fostering balance, variety, and modeling healthy eating behaviors. Such programs can be delivered through community-based workshops, pediatric clinics, or digital platforms, ensuring accessibility for diverse caregiver populations.

## 5. Conclusions

Guided by a framework derived from the TPB, this study provides a comprehensive examination of how demographic characteristics, dietary beliefs, and intentions influence the feeding practices of primary caregivers of preschool-aged children. The findings highlight the complex interplay between cognitive and sociodemographic factors in shaping caregivers’ feeding behaviors, offering valuable insights into the determinants of early childhood dietary practices. Importantly, caregiver intentions to provide a healthy diet and behavioral beliefs emerged as a significant predictor of multiple feeding practices, reinforcing the need for targeted interventions that enhance caregivers’ motivation, self-efficacy, and dietary knowledge in fostering positive feeding practices. While previous research has primarily focused on demographic influences, this study uniquely integrates cognitive factors to better explain variations in feeding practices. The results underscore the necessity of moving beyond traditional demographic assessments to include caregivers’ beliefs and intentions when designing interventions aimed at the prevention of childhood obesity. Additionally, the findings suggest that interventions should be tailored to account for caregiver-specific factors, such as perceived weight status, income level, and level of education, which were found to influence feeding behaviors.

Overall, this study emphasizes the critical role of caregivers in shaping children’s dietary habits and provides a foundation for the development of targeted, evidence-based interventions. By integrating both cognitive and demographic determinants, future interventions and public health initiatives can more effectively support caregivers in adopting healthier feeding practices, ultimately contributing to the prevention of childhood obesity and the promotion of lifelong healthy eating habits.

## Figures and Tables

**Table 1:** Demographic characteristics (background) of primary caregivers of preschoolers.

Demographic Characteristics of Primary Caregivers (n = 146)	M ± SD (Range)
Age	35.4 ± 6.2 (25–69)
Body Mass Index (BMI)	32.2 ± 9.4 (17.3–78.8)
Years of Education	16.4 ± 4.1 (5–48)
**Categorical Variables n (%)**	
Gender	
Female	140 (95.9)
Male	6 (4.1)
Other	0 (0)
Biological Sex	
Female	140 (95.9)
Male	6 (4.1)
Number of Children Aged between 3 and 5	
1	119 (81.5)
2	21 (14.4)
3	3 (2.1)
4 or more	3 (2.1)
Smoking Status	
Never Smoking	123 (84.2)
Yes, regular smoker	10 (6.8)
Quit or Past Smoking	12 (8.2)
Other (Not regular once in a while)	1 (0.7)
Race	
White or Caucasian	113 (77.4)
Non-White	33 (22.6)
Ethnicity	
Not Hispanic or Latino	128 (87.7)
Hispanic or Latino	18 (12.3)
Marital Status	
Never Married	16 (11.0)
Married	112 (76.7)
Divorced	8 (5.5)
Member of an unmarried couple	10 (6.8)
Working Status	
Full-time, 35 h or more	50 (34.2)
Part-time, less than 35 h	29 (19.9)
Self-employed in your own business	28 (19.2)
Unemployment compensation	12 (8.2)
Retired	5 (3.4)
Refuse to answer	22 (15.1)
Total Household Income per year (USD)	
Less than 10,000	6 (4.1)
10,000–19,999	7 (4.8)
20,000–29,999	10 (6.8)
30,000–39,999	13 (8.9)
40,000–49,999	11 (7.5)
50,000–59,999	15 (10.3)
60,000–69,999	14 (9.6)
70,000–79,000	14 (9.6)
80,000–89,999	8 (5.5)
90,000–99,999	12 (8.2)
More than 100,000	36 (24.7)
Perceived Household Income	
Comfortable	66 (45.2)
Just have enough to make ends meet	69 (47.3)
Do NOT have enough to make ends meet	11 (7.5)
Perceived Concern about Current Weight	
NO	64 (43.8)
Yes	82 (56.2)
Perceived Weight Status	
Underweight	1 (0.7)
Normal Weight	39 (26.7)
Overweight	83 (56.8)
Obese	21 (14.4)
Unable to perceive weight (do not know)	2 (1.4)
Following a Diet	
No	117 (80.1)
Yes	29 (19.9)
Relationship with your child	
Biological Mother	129 (88.4)
Adoptive mother	6 (4.1)
Biological Father	6 (4.1)
Stepmother	2 (1.4)
Grandmother	3 (2.1)
Perceived Weigh of Their Child	
Underweight	14 (9.6)
Normal Weight	129 (88.4)
Overweight	2 (1.4)
Obese	1 (0.7)
Concern about their Child’s Weight	
No	133 (91.1)
Yes	9 (6.2)
Unsure	4 (2.7)

**Table 2:** Demographic characteristics significantly associated with primary caregivers’ feeding practices.

Demographic Characteristic	Feeding Practice Subscale		*p*-Value
		**Pearson Correlation Coefficient (r)**	
**Age**			
Primary caregivers	Teaching about nutrition	−0.2	0.01 **
Children	Restriction for Health	0.2	0.04 *
**BMI**			
Primary caregivers	Emotion regulation	−0.2	0.04 *
Children	Restriction for weight control	0.2	0.003 **
**Years of education**	Emotion regulation	0.2	0.0 ***
	Encourage balance and variety	−0.2	0.04 *
	Restriction for Health	−0.2	0.004 **
		***t*-Test**	
**Sex**			
Male caregivers	Restriction for weight control	−3.9	<0.001 ***
Non-White Race	Monitoring	−2.2	0.03 *
	Restriction for Health	−2.3	0.02 *
	Restriction for weight control	−2.2	0.03 *
Following a Diet- Yes	Environment	2.8	0.006 **
		**ANOVA (F-value)**	
Marital Status	Environment	3.3	0.023 *
	Restriction for Health	3.9	0.01 *
Smoking Status	Involvement	2.9	0.04 *
Working Status	Involvement	3.3	0.008 **
	Restriction for Health	3.5	0.005 **
Relationship to children (Mother, Father, Grandson)	Food as reward	2.5	0.046 *
	Restriction for weight control	8	<0.001 ***
Perceived Household Income	Modeling	3.8	0.02 *
Concern about Child’s weight	Environment	5.6	0.005 **
Perceived Weight	Control	4.2	0.003 **
	Emotion regulation	2.5	0.04 *
Perceived children weight	Environment	4.03	0.009 **
	Restriction for weight control	3.7	0.01 *

* *p* < 0.05. ** *p* < 0.01. *** *p* < 0.001.

**Table 3:** Results of hierarchical multiple linear regression analysis—significant predictors of each feeding practice subscale.

Dependent Variable	R²	Adjusted R^2^	Δ R²	Predictor Variable (s)	Standardized β Coefficients	*p*-Value
Control	0.202	0.162	0.026	Perceived weight of primary caregivers:		<0.001
				Normal weight	0.202	0.012 *
				Unable to perceive weight (don’t know)	−0.198	0.011 *
				Behavioral Beliefs	−0.196	0.017 *
				Intentions to provide a healthy diet	−0.167	0.036 *
Emotion Regulation	0.161	0.118	0.052			0.004
				Hindrances control belief	0.2936	0.004 **
Encourage balance and variety	0.284	0.253	0.123			<0.001
				Years of Education	−0.172	0.020 *
				Intention to provide a healthy diet	0.374	<0.001 ***
Environment	0.204	0.145	0.024			0.047
				Following a diet	−0.164	0.049 *
				Divorce marital status	−0.186	0.020 *
				Behavioral Beliefs	0.159	0.047 *
Food as a Reward	0.109	0.077	0.043			0.010
				Being Grandmothers	−0.212	0.009 **
				Behavioral Beliefs	0.208	0.010 **
Involvement	0.24	0.19	0.087			<0.001
				Another smoking status	−0.157	0.046 *
				Behavioral Beliefs	0.301	<0.001 ***
Modeling	0.280	0.244	0.041			0.006
				Comfortable Income	0.168	0.030 *
				Behavioral beliefs	0.23	0.008 **
				Hinderance control beliefs	−0.218	0.005 **
				Intentions to provide a healthy diet	0.216	0.006 **
Monitoring	0.164	0.14	0.03			0.025
				Facilitators control beliefs	0.222	0.009 **
				Intentions to provide a healthy diet	0.178	0.025 *
Pressure	0.043	0.037	0.043			0.012
				Behavioral beliefs	0.209	0.012 *
Restriction for health	0.309	0.241	0.076			0.001
				Child age	0.202	0.008 **
				Employment status—not working	0.248	0.003 **
				Hinderance control beliefs	0.252	0.002 **
Restriction for weight control	0.294	0.236	0.043			0.019
				Stepmother	0.210	0.014 *
Teaching about nutrition	0.169	0.145	0.031			0.023
				Age of the primary caregivers	−0.169	0.032 *
				Behavioral beliefs	0.283	0.001 **
				Intentions to provide a healthy diet	0.182	0.023 *

* *p* < 0.05. ** *p* < 0.01. *** *p* < 0.001.

## Data Availability

The data supporting the reported results of this study are not publicly available due to ethical restrictions and the protection of participant confidentiality. Access to the data may be considered on a case-by-case basis and can be obtained by contacting the corresponding author at oudatqh@mail.uc.edu.

## Supplementary Materials

The following supporting information can be downloaded at: https://www.mdpi.com/article/10.3390/children12020226/s1, Table S1: The correlation between dietary beliefs and intentions with the feeding practice subscales of primary caregivers.
