# Supplementary material for: The Factors Influencing Feeding Practices of Primary Caregivers of Preschoolers: A Theory-Based Cross-Sectional Study
Source: Children (Basel). 2025 Feb 13;12(2):226. doi: 10.3390/children12020226 (PMC11854846; doi:10.3390/children12020226)
Supplement: Supplementary file 1 [file children-12-00226-s001.zip › children-3463599-supplementary.pdf]

**Table S1**. Correlation Between Dietary Beliefs and Intentions with the Feeding Practice Subscales of Primary Caregivers.

| Variables | **Control** | | **Emotion regulation** | | **Encourage balance and variety** | | **Environment** | | **Food as reward** | | **Involvement** | | **Modeling** | | **Monitoring** | | **Pressure** | | **Restriction for Health** | | **Restriction for weight control** | | **Teaching about nutrition** | |
|---|---|---|---|---|---|---|---|---|---|---|---|---|---|---|---|---|---|---|---|---|---|---|---|---|
| | **r** | **sig** | **r** | **sig** | **r** | **sig** | **r** | **sig** | **r** | **sig** | **r** | **sig** | **r** | **sig** | **r** | **sig** | **r** | **sig** | **r** | **sig** | **r** | **sig** | **r** | **sig** |
| **Dietary Beliefs** | | | | | | | | | | | | | | | | | | | | | | | | |
| Behavioral | -0.24** | 0.003 | 0.04 | 0.66 | 0.23** | 0.005 | 0.172* | 0.038 | 0.211* | 0.011 | 0.08 | 0.34 | 0.26** | 0.002 | 0.23** | 0.006 | 0.21* | 0.012 | 0.21* | 0.012 | 0.17* | 0.040 | 0.330*** | <0.001 |
| Normative | -0.16 | 0.06 | 0.04 | 0.63 | 0.20* | 0.014 | -0.033 | 0.7 | 0.107 | 0.199 | 0.15 | 0.08 | 0.24** | 0.003 | 0.088 | 0.29 | 0.05 | 0.53 | -0.01 | 0.902 | -0.03 | 0.69 | 0.19* | 0.03 |
| Control | | | | | | | | | | | | | | | | | | | | | | | | |
| Facilitators | -0.174* | 0.04 | -0.04 | 0.64 | 0.217** | 0.008 | 0.05 | 0.56 | -0.027 | 0.75 | 0.32*** | <0.001 | 0.27*** | <0.001 | 0.32** | <0.001 | 0.04 | 0.59 | 0.09 | 0.24 | 0.26** | 0.002 | 0.08 | 0.29 |
| Hindrances | 0.12 | 0.14 | 0.226** | 0.006 | -0.17* | 0.045 | 0.11 | 0.20 | 0.24** | 0.004 | -0.13 | 0.117 | -0.21* | 0.012 | -0.07 | 0.43 | 0.004 | 0.96 | 0.217** | 0.008 | 0.06 | 0.46 | 0.06 | 0.49 |
| **Intentions to Provide a Healthy Diet** | -0.20* | 0.015 | -0.16 | 0.06 | 0.44*** | <0.001 | 0.009 | 0.91 | -0.110 | 0.18 | 0.078 | 0.35 | 0.33*** | <0.001 | 0.24** | 0.003 | 0.06 | 0.48 | 0.02 | 0.80 | 0.04 | 0.68 | 0.22** | 0.007 |

*$p < 0.05$. **$p < 0.01$. ***$p < 0.001$
